# Complete protection of the BALB/c and C57BL/6J mice against Ebola and Marburg virus lethal challenges by pan-filovirus T-cell epigraph vaccine

**DOI:** 10.1371/journal.ppat.1007564

**Published:** 2019-02-28

**Authors:** Md Niaz Rahim, Edmund G. Wee, Shihua He, Jonathan Audet, Kevin Tierney, Nathifa Moyo, Zara Hannoun, Alison Crook, Andrea Baines, Bette Korber, Xiangguo Qiu, Tomáš Hanke

**Affiliations:** 1 National Microbiology Laboratory, Public Health Agency of Canada, Winnipeg, MB, Canada; 2 Department of Medical Microbiology, University of Manitoba, Winnipeg, MB, Canada; 3 The Jenner Institute, Nuffield Department of Medicine, University of Oxford, Oxford, United Kingdom; 4 Los Alamo National Laboratory, Theoretical Biology and Biophysics, Los Alamos, New Mexico, United States of America; 5 The New Mexico Consortium, Los Alamos, New Mexico, United States of America; 6 International Research Center for Medical Sciences, Kumamoto University, Kumamoto, Japan; Division of Clinical Research, UNITED STATES

## Abstract

There are a number of vaccine candidates under development against a small number of the most common outbreak filoviruses all employing the virus glycoprotein (GP) as the vaccine immunogen. However, antibodies induced by such GP vaccines are typically autologous and limited to the other members of the same species. In contrast, T-cell vaccines offer a possibility to design a single pan-filovirus vaccine protecting against all known and even likely existing, but as yet unencountered members of the family. Here, we used a cross-filovirus immunogen based on conserved regions of the filovirus nucleoprotein, matrix and polymerase to construct simian adenovirus- and poxvirus MVA-vectored vaccines, and in a proof-of-concept study demonstrated a protection of the BALB/c and C57BL/6J mice against high, lethal challenges with Ebola and Marburg viruses, two distant members of the family, by vaccine-elicited T cells in the absence of GP antibodies.

## Author summary

Development of an effective vaccine against filovirus outbreaks is an important public health aim. Here, we demonstrate the principle that cellular responses can not only protect two strains of mice against a high lethal virus challenge of 1000 LD_50_ in the absence of glycoprotein antibodies, but a single epigraph T–cell vaccine can do so against distant members of the filovirus family, EBOV and MARV. This suggests a possibility that this candidate vaccine also protects against other known as well as yet unencountered viruses of the filovirus family; it is a pan-filovirus vaccine.

## Introduction

The family *Filoviridae* includes 5 distinct viruses in the Ebolavirus genus: Zaire Ebola virus (EBOV), Sudan virus (SUDV), Reston virus (RESTV), Tai Forest virus (TAFV), and Bundibugyo virus (BDBV); 2 viruses in the Marburg-virus genus: Marburg virus (MARV) and Ravn virus (RAVV); and 1 virus in the Cuevavirus genus: Lloviu virus (LLOV). The first identified filovirus disease was caused by MARV and occurred in Europe in 1967. Since then, there have been over 50 recorded zoonotic outbreaks causing hemorrhagic fevers in humans and non-human primates with 90% fatality rates [1, 2]. There is no vaccine or drug licensed against any member of the filovirus family. Thus, development of an effective vaccine is of great importance for public health in Africa, where outbreaks occur periodically, as well as for the rest of the world.

At least seven vaccine platforms vectored by human and simian (chimpanzee) adenoviruses HAdV-5, HAdV-26, ChAdV-3, vesicular stomatitis virus (VSV), human cytomegalovirus, modified vaccinia virus Ankara (MVA), plasmid DNA, subunit proteins and virus-like particles have been tested in nonhuman primates (NHPs) and encouraging results were obtained with two candidates, replicating VSV-ZEBOV (EBOV) and non-replicating ChAd3-ZEBOV, showing a single dose efficacy against EBOV challenge [3, 4]. However, before the 2013 epidemic, only one vaccine reached phase 1 trial in humans and was abandoned. Facing the 2013 epidemic, the most promising vaccines were moved to clinical trials [5–10] and one, rVSV-ZEBOV reported efficacy in a human phase 3 trial [6]. During the 2018 Ebola outbreak in the Democratic Republic of Congo, death toll was reduced to 29 due to a number of factors; the rVSV-ZEBOV vaccine was experimentally deployed, but no data indicated its contribution to the reduced outbreak.

Most of the above efforts focus on EBOV, because this virus is historically the most frequent cause of filovirus outbreaks, and all employ the virus glycoprotein (GP). While there is a high degree of conservation in the GP within one species, so that, for example, antibody responses to EBOV vaccine would likely cross-react with other EBOV outbreak variants, protection against other filoviruses by the current vaccines will be very low [11]. Indeed, rVSV-ZEBOV induced 50% cross-protection for SUDV [12] and protection against other more distant viruses of the filovirus family would likely be much lower and require a multi-species vaccine [13].

An ideal vaccine should be effective not only against the currently prioritized outbreak species, but across all variants of the 8 distinctive filovirus members and provide a degree of protection even against the likely existing, but as yet unencountered species. Induction of CD8^+^ T-cells provides such an opportunity. The FILOcep1&2 vaccines constructed here aim to induce protective T-cell responses against viruses across the filovirus family. While the four most conserved regions of the filovirus family were identified and the theoretical corresponding epigraph regions were computed previously [11], in the present work, we describe construction of the candidate pan-filovirus T-cell four-component vaccine vectored by simian adenovirus and poxvirus MVA, demonstrate their broad immunogenicity in the BALB/c and C57BL/6J strains of mice and report a solid protection of mice by vaccination from highly lethal EBOV and MARV experimental challenges. This protection was mediated solely by T-cell responses in the absence of GP-specific antibodies. The possible role of this vaccine in the preparedness for the future filovirus outbreaks as well as its use for treating residual infection are discussed.

## Results

### Construction of the FILOcep1&2 vaccines

**Fig 1:**
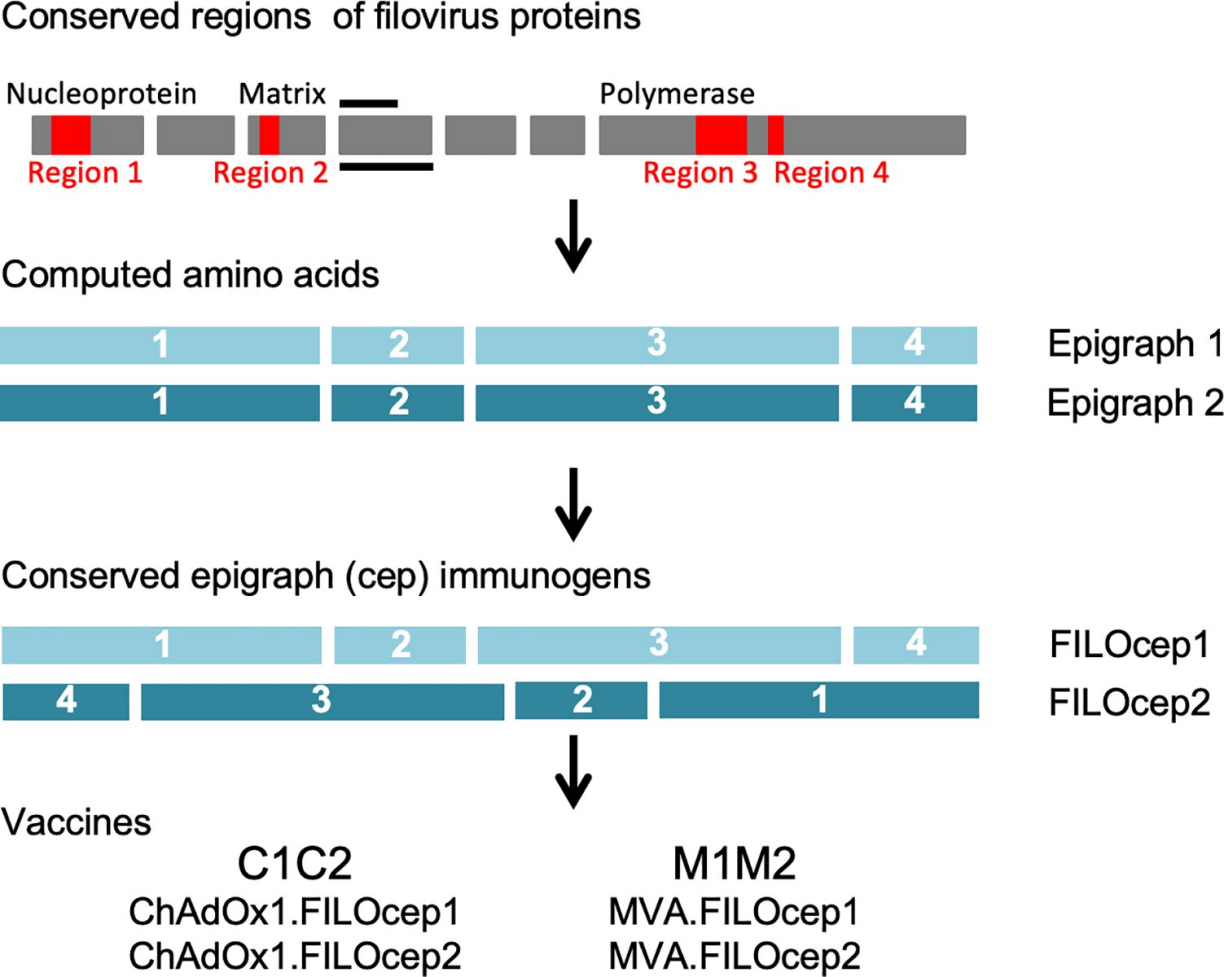
The FILOcep1&2 vaccine design. Conserved regions of the filovirus proteome (red) are the most similar parts of proteins common across the eight virus species of the filovirus family. These regions were identified by amino acid alignment of all known filovirus isolates in the database. An algorithm called Epigraph computed bi-valent amino acid sequences (epigraph 1 and epigraph 2), which complement each other and are used together in a vaccine to optimize match of potential T-cell epitopes between the vaccine and all input filovirus species [11]. For the FILOcep1 and FILOcep2 epigraphs, the four regions 1, 2, 3 and 4 are 280 (nucleoprotein 131–410), 123 (matrix 71–193), 315 (RNA polymerase 540–854) and 109 (RNA polymerase 952–1060) amino acid long, respectively, and were arranged into different orders to minimize potential induction of T cells recognizing irrelevant (non-viral) newly generated epitopes across the regional junctions. Synthetic ORF coding for these two proteins each 827 amino acid in length were inserted into engineered replication-deficient simian (chimpanzee) adenovirus ChAdOx1 and replication-deficient poxvirus MVA to generate four components of the vaccine abbreviated C1, C2, M1 and M2.

The FILOcep1&2 vaccines aim to induce protective T-cell responses against viruses across the filovirus family. This is achieved by targeting the most similar, structurally and functionally conserved regions among the virus proteomes, and maximizing the match of the vaccine to all potential 9-mer T-cell epitopes (PTE) within these regions by computing bi-valent Epigraph sequences [11]. Epigraphs are the next generation of the pluri-valent mosaic design [14] aiming to maximize the coverage of a diverse, variable population of pathogens by bioinformatics-assisted computed amino acid sequences. The main improvement over mosaic is that epigraphs “walks” through the protein sequence and ensures that all PTE sequences used occur in the natural isolates present in the starting database. The rationale of the immunogen design is as follows. The best match is ensured with the EBOV species, which historically seeded the most outbreaks. The immunogens still have an excellent match to the other common outbreak species SUDV and MARV, and within the conserved regions maintain a good match to all other known filovirus PTEs [11]. Overall, there is a minimum of 8/9-amino acid match within a PTE to 80% filovirus isolates. Each of the four regions of epigraph 1 and epigraph 2 differ in about 10% amino acids, include a span of minimum 100 amino acids, together total 827 amino acids, and ensure broad representation of human leukocyte antigens (HLAs) for the restricted epitopes. To decrease potential induction of strong irrelevant CD8^+^ T cells recognizing new and, therefore, irrelevant non-viral epitopes generated by joining two adjacent regions together, the four conserved filovirus regions are assembled in two unique orders: 1-2-3-4 in FILOcep1 and 4-3-2-1 in FILOcep2 (Fig 1).

The FILOcep1&2 immunogens were delivered to the cells of the immune system employing non-replicating engineered chimpanzee adenovirus ChAdOx1 and non-replicating poxvirus MVA as vaccine vectors [15]. The combination of these heterologous vectors has been shown to induce robust CD8^+^ T-cell responses in human volunteers for other indications [5, 16, 17]. Here, synthetic open-reading frames coding for FILOcep1 and FILOcep2 were inserted into the vector genomes to be administered in a four-component vaccine regimen, whereby the ChAdOx1.FILOcep1 + ChAdOx1.FILOcep2 vaccines were used together as a prime and MVA.FILOcep1 + MVA.FILOcep2 were used together as a boost (Fig 1).

### Optimization and characterization of vaccine-elicited T-cell responses

**Fig 2:**
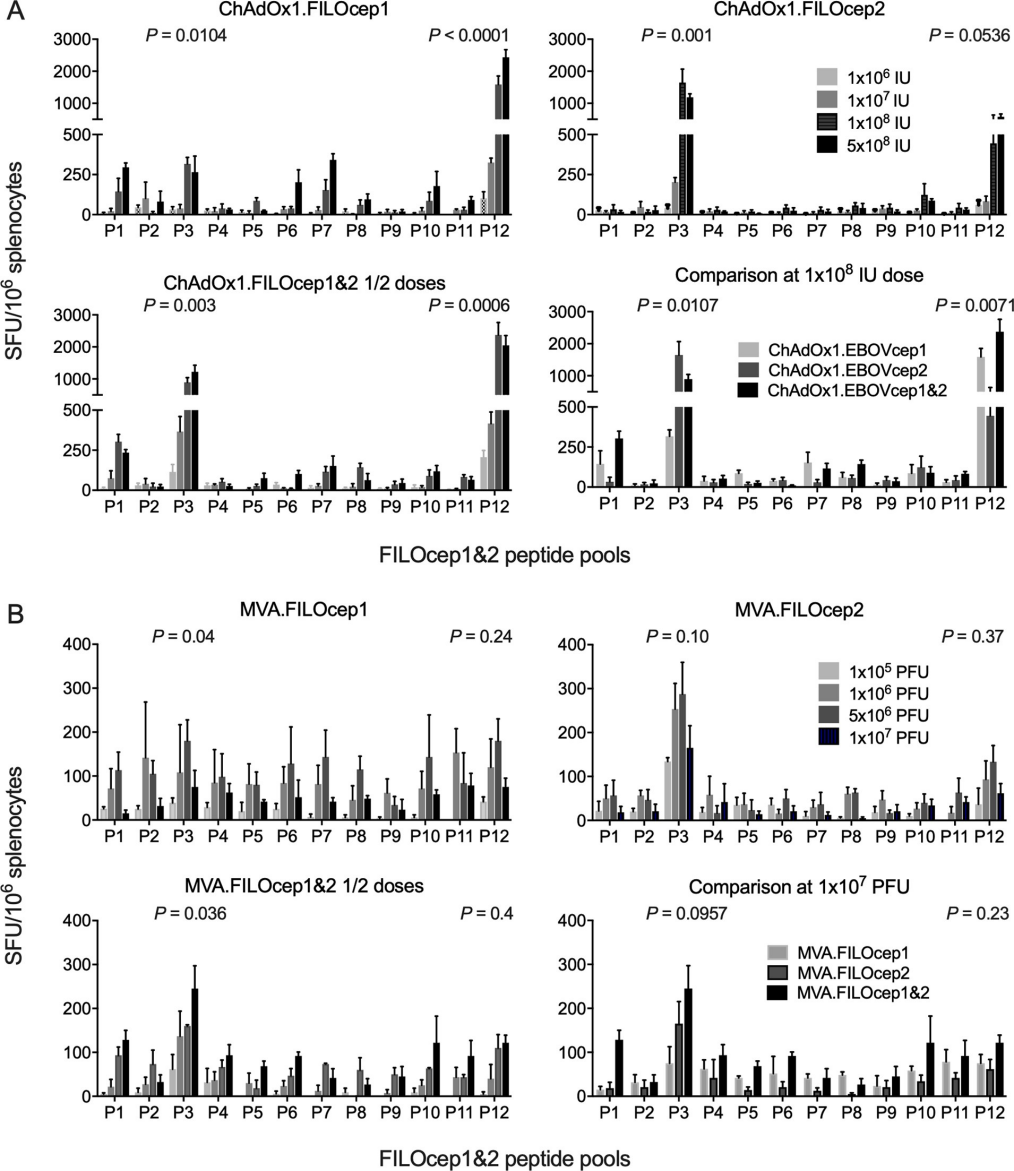
Dose finding for rMVA and rChAdOx1 vaccines. Groups of the BALB/c mice were administered intramuscularly increasing doses of 1x10^6^, 1x10^7^, 1x10^8^ and 5x10^8^ infectious units (IU) of individual ChAdOx1.FILOcep1 and ChAdOx1.FIOcep2 vaccines and their half-dose combinations A), or 1x10^5^, 1x10^6^, 5x10^6^ and 1x10^7^ plaque-forming units (PFU) of MVA.FILOcep1 and MVA.FILOEcep2 vaccines and their combined half-doses B), and the frequencies of the vaccine-elicited filovirus-specific T cells in the spleen were assessed 9 days after vaccination in an IFN-γ ELISPOT assay using 12 pools of FILOcep1&2-derived 15-mer peptides overlapping by 11 amino acids and spanning the full length of both immunogens. T-cell epitope variant peptide pairs were used together in pools to allow addition of pool-detected frequencies for overall magnitude of the anti-FILOcep1&2 responses. Data are shown as median (range), n = 3. Kruskal-Wallis test was used to determine the significance of variation among individual doses/vaccinations for immunodominant peptide pools P3 and P12 and the *P* values are shown above.

**Fig 3:**
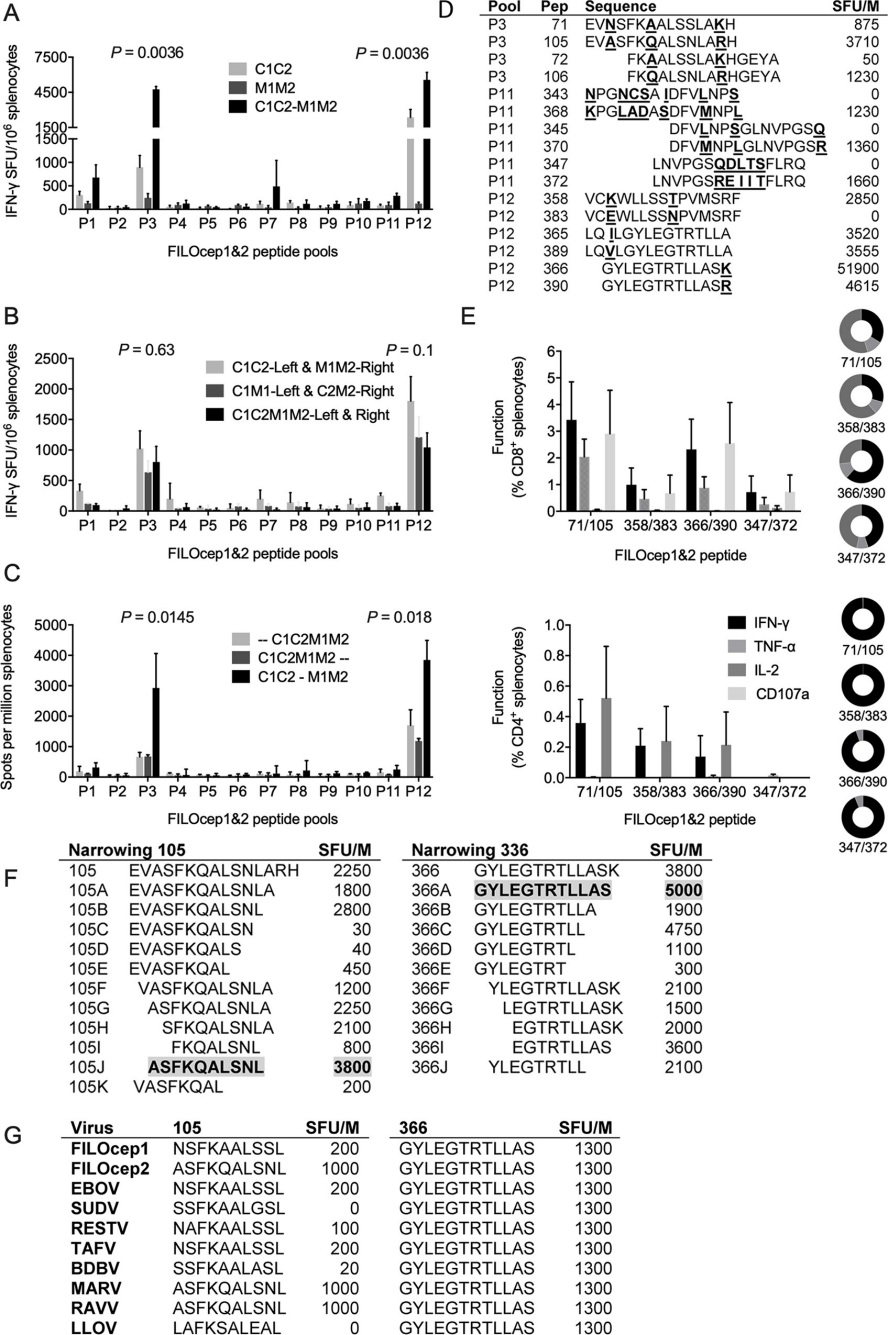
Optimization of the regimen and characterization of FILOcep1&2 vaccine-elicited responses in the BALB/c mice. Groups of mice were vaccinated A) with either the two adenovirus or two poxvirus components alone and compared to their C1C2 prime-M1M2 boost combination, B) exploring various vaccine distributions between two anatomical sites (hind legs) and compared to C1C2-M1M2, C) administering all 4 vaccine components on the same day and assaying at 4 weeks or 1 week later compared to C1C2-M1M2. The induced T cells were analyzed employing 12 FILOcep1&2 peptide pools in an IFN-γ ELISPOT assay. For A), B) and C), Kruskal-Wallis test was used to determine the significance of variation among regimens for immunodominant peptide pools P3 and P12 and the *P* values are shown above. D) Using the most efficient C1C2-M1M2 regimen, all 390 15-mer peptides were tested individually in an IFN-γ ELIPSPOT assay (S1 Fig.) and the strongest peptide pairs from that scan are listed, whereby SFU/M gives the frequencies of responding splenocytes per million. E) The 4 most immunodominant 15-mer peptide pairs used to characterize the functionality of vaccine-elicited CD8^+^ and CD4^+^ T cells, whereby the plurifunctionality of cells expressing 1 (black), 2 (light gray) and 3 (dark gray) cytokines/functions simultaneously are given as pie charts. Data in A), B), C) and E) are shown as median (range), n = 4. F) Two immunodominant CD8^+^ T-cell epitopes in 15-mers 105 and 336 were narrowed to their optimal length using IFN-γ ELISPOT assay with the frequencies of responding T cells on the right. G) In a separate immunization experiment, optimal-length variant epitopes derived from the 8 members of the filovirus family were assessed for recognition by C1C2-M1M2-induced T cells.

We optimized and characterized the vaccine-elicited T-cell responses in the BALB/c mice (H-2^d^). For each vaccine component individually and two epigraphs together, four escalating doses were administered intramuscularly. The frequencies of FILOcep1&2-specific T cells were determined in an IFN-γ ELISPOT assay employing 12 pools of variant peptide pairs derived from the two FILOcep1 and FILOcep2 epigraphs. Thus, doses ranging from 10^6^ to 5x10^8^ infectious units (IU) were assessed for ChAdOx1.FILOcep1 (C1) and ChAdOx1.FILOcep2 (C2) individually and for half-doses together as C1C2, and the dose of 1x10^8^ IU was chosen for further vaccinations (Fig 2A). For MVA.FILOcep1 (M1), MVA.FILOcep2 (M2) and two half-doses of M1M2, a range from 1x10^5^ to 1x10^7^ plaque-forming units (PFU) was tested and 10^7^ PFU was chosen for further experiments (Fig 2B). Broadly specific responses against 8 pools with higher that 50 SFU/10^6^ splenocytes and dominant pools P3 and P12 were induced, which summed across all 12 pools for the combined C1C2 and M1M2 deliveries to median of 4207 and 1109 SFU/10^6^ splenocytes, respectively. Next, we determined that C1C2 was synergistically boosted with M1M2 totalling median 12495 SFU/10^6^ splenocytes (Fig 3A). The most potent was a combination of C1C2 delivered into one site and M1M2 into another site over the mixed C1M1 and C2M2 administration (Fig 3B). Administration of all four vaccine components at the same time was much less potent than heterologous C1C2 prime and M1M2 boost separated by 3 weeks (Fig 3C). In the BALB/c mice, we mapped highly stimulatory 15-mer peptides (S1 Fig and Fig 3D) and used their pairs, one from each epigraph, to demonstrate induction of plurifunctional IFN-γ, TNF-α, IL-2 and CD107a responses. CD8^+^ T cells produced mainly IFN-γ, TNF-α and degranulated (CD107a) concurring with their cytolytic capacity, while CD4^+^ T cells produced IFN-γ, and IL-2 (Fig 3E). Between 27% to 61% of CD8^+^ T cells produced 3 functions in parallel, while CD4^+^ T cells were mainly monofunctional. We narrowed down the two most immunodominant CD8^+^ T-cell responses in peptides 105 and 336 to ASFKQALSNL (AL10) and GYLEGTRTLLAS (GS12), respectively (Fig 3F). The optimal length of these epitopes present in the two vaccine epigraphs were compared to the sequences across the filovirus family. Epitope variants N/ASFKQALSNL in FILOcep1 and FILOcep2 matched EBOV and MARV, respectively, and differed for several other filoviruses with the strongest ASFKQALSNL (MARV and RAVV) yielding 1000 SFU/10^6^ splenocytes and SSFKAALGSL (SUDV) and LAFKSALEAL (LLOV) not recognized at all. In contrast, GS12 was conserved across the entire family and strongly recognized at 1300 SFU/10^6^ splenocytes (Fig 3G).

### Complete protection against highly lethal doses of EBOV and MARV in the BABL/c mice

**Fig 4:**
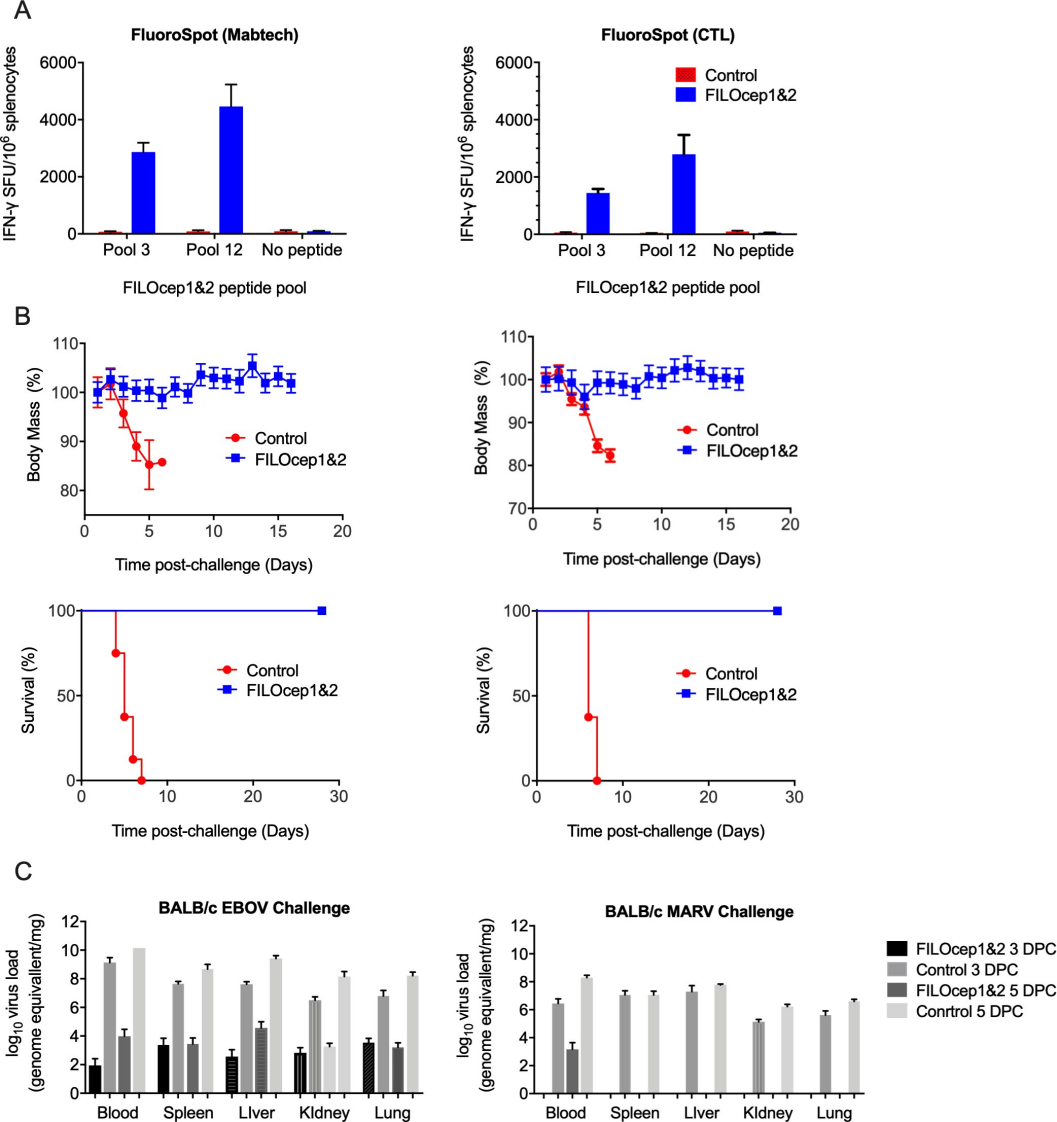
Complete protection of the BALB/c mice against Ebola and Marburg virus challenges by FILOcep1&2 vaccination. BALB/c mice were immunized with the candidate FILOcep1&2 vaccines or control vaccines expressing irrelevant eGFP, challenged by Ebola and Marburg viruses as shown in Table 1. A) Induction of filovirus-specific T cells was confirmed using Mabtech (left) and Cellular Technology Limited (CTL; right) IFN-γ ELSIPOT assay kits using two immunodominant peptide pools P3 and P12 on day 28. Frequencies are shown as median (range), n = 4. B) Eight mice in the FILOcep1&2 (blue) and 8 in the control eGFP (red) groups were challenged with 1000 LD_50_ of either mouse-adapted EBOV (Mayinga; left) or 1000 LD_50_ mouse-adapted MARV (Angola; right) virus on day 35 and the animals were daily measured for their body mass till day 14 post challenge (top) and survival till day 28 post challenge (bottom). The *P* values for survival used the Log-rank (Mantel-Cox) Test, n = 8. C) The EBOV and MARV viruses were quantified in various tissues on 3 and 5 days after the challenge (DPC). Data are shown as median (range), n = 4 per group.

Next, we set out to assess the protective efficacy of the vaccine-elicited T cells, in our case in the absence of any GP-specific antibody, against two distant filoviruses, EBOV and MARV. Using the best regimen of the 4 vaccine components identified above, groups of 20 BALB/c mice received either the FILOcep1 and FILOcep2 vaccines or control eGFP vaccines, the latter expressing enhanced green fluorescent protein (eGFP) as an irrelevant protein with no homology to the filovirus family (Table 1). Four animals in each group were sacrificed 1 week after they received rMVA and, employing two different commercial IFN-γ ELISPOT kits, high frequency T cells specific for the FILOcep1&2 immunogens were detected in animals receiving the test vaccines, while no FILOcep1&2-specific responses were induced by the control eGFP vaccines (Fig 4A). This confirmed compatible immunopotency between the Oxford and Winnipeg laboratories. Of the remaining 16 animals in each group, 8 were exposed to a lethal challenge with 1000 LD_50_ of mouse-adapted EBOV (Mayinga) [18] and 8 with 1000 LD_50_ of mouse-adapted MARV (Angola) [19] 4 weeks post vaccination and their body mass was recorded daily. While all the animals in the control group started losing mass precipitously and either died or had to be euthanized between days 4 and 6 post challenge, all the FILOcep1&2 vaccine recipients maintained normal body mass and survived till the end of the scheduled protocol on day 29 post challenge (Fig 4B). In the repeat experiment, mice were sacrificed 3 and 5 days after challenge and the EBOV and MARV genomes were quantified in the blood, spleen, liver, kidneys and lungs. For EBOV, between 4 and 6 log_10_ fewer genomes per mg of tissue were found in FILOcep1&2 vaccinated mice, while for MARV, virus was only detected in FILOcep1&2 vaccine recipients in the blood at 1000 genomes/mg on day 5 after challenge (Fig 4C). We conclude that the T-cell responses induced by the ChAdOx1.FILOcep1 + ChAdOx1.FILOcep2 prime-MVA.FILOcep1 + MVA.FILOcep2 boost regimen protected the BALB/c mice from both the EBOV and MARV lethal challenges and did so in the absence of glycoprotein antibodies.

**Table 1:** Design of the experimental challenges.

	Day 0	Day 21	Day 28	Day 35	Up to day 64
**Vaccine**	**ChAdOx1.FILOcep1**	**MVA.FILOcep1**	IFN-γ ELISPOT	**EBOV** challenge1000 LD_50_	
	5x10^7^ IU i.m.	5x10^6^ PFU i.m.	assay	8 animals	Monitoring
20	+	+	4 animals		
animals	**ChAdOx1.FILOcep2**	**MVA.FILOcep2**		**MARV** challenge1000 LD_50_	
	5x10^7^ IU i.m.	5x10^6^ PFU i.m.		8 animals	
**Contro**l				**EBOV** challenge1000 LD_50_	
	**ChAdOx1.eGFP**	**MVA.eGFP**	IFN-γ ELISPOT	8 animals	Monitoring
20	1x10^8^ IU i.m.	1x10^7^ PFU i.m.	assay		
animals			4 animals	**MARV** challenge1000 LD_50_	
				8 animals	

This design was used in two BALB/c and one C57BL/6J experimental challenges.

### Immunopotency and protection against highly lethal doses of EBOV and MARV in the C57BL/6J mice

**Fig 5:**
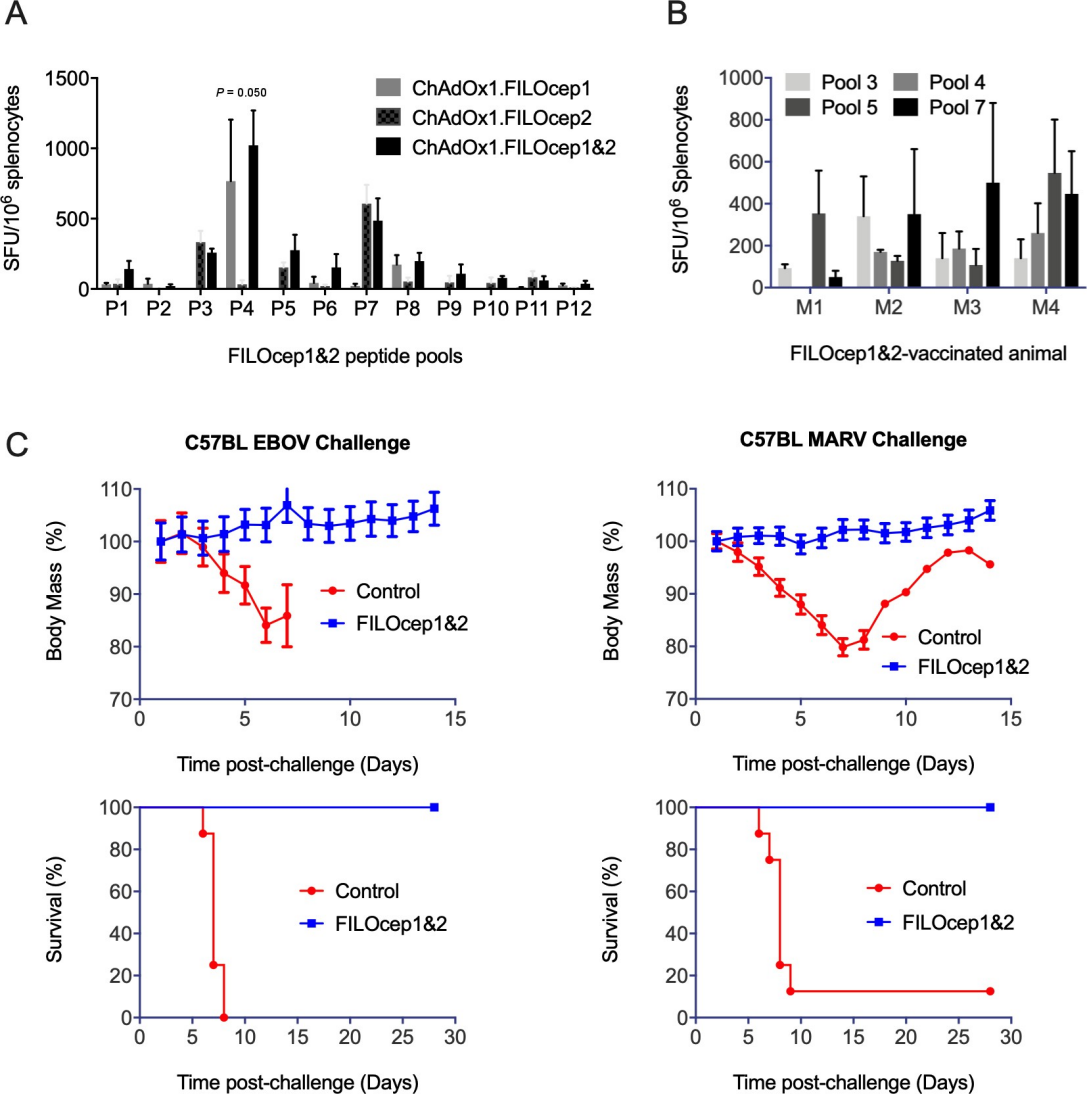
Complete protection of the C57BL/6J mice against Ebola and Marburg virus challenges by FILOcep1&2 vaccination. A) A group of the C57BL/6J mice was vaccinated using the C1C2 and M1M2 3 weeks apart and the pattern of immunodominance for the 12 FILOcep1&2 peptide pools was determined in an IFN-y ELISPOT assay in the Oxford laboratory 1 week later (n = 4). B) In the Winnipeg laboratory, groups of the C57BL/6J mice were immunized with the candidate FILOcep1&2 vaccines or control vaccines expressing irrelevant eGFP and challenged by Ebola and Marburg viruses on day 35 (Table 1). Kruskal-Wallis test was used to determine the significance of variation among regimens for immunodominant peptide pool P4 and the *P* value is shown above. B) Four mice were killed and the induction of filovirus-specific T cells was confirmed in an IFN-γ ELSIPOT assay kits using the 4 immunodominant peptide pools P3, P4, P5 and P7. C) Eight mice in the FILOcep1&2 (blue) and 8 in the control eGFP (red) groups were challenged with 1000 LD_50_ of either mouse-adapted EBOV (Mayinga; left) or 1000 LD_50_ mouse-adapted MARV (Angola; right) virus and animals’ body mass was measured daily till day 14 post challenge (top) and survival was monitored until day 28 post challenge (bottom). The *P* values for survival used the Log-rank (Mantel-Cox) Test, n = 8.

We also assessed the breadth of T-cell responses induced in the C57BL/6J strain of mice (H-2^b^). Groups of mice were immunized with either FILOcep1, FILOcep2 or combined half-doses of both epigraphs and a pattern on immunodominance was observed distinct from that in the BALB/c mice with the strongest peptide pools P3, P4, P5 and P7 (Fig 5A).

**Fig 6:**
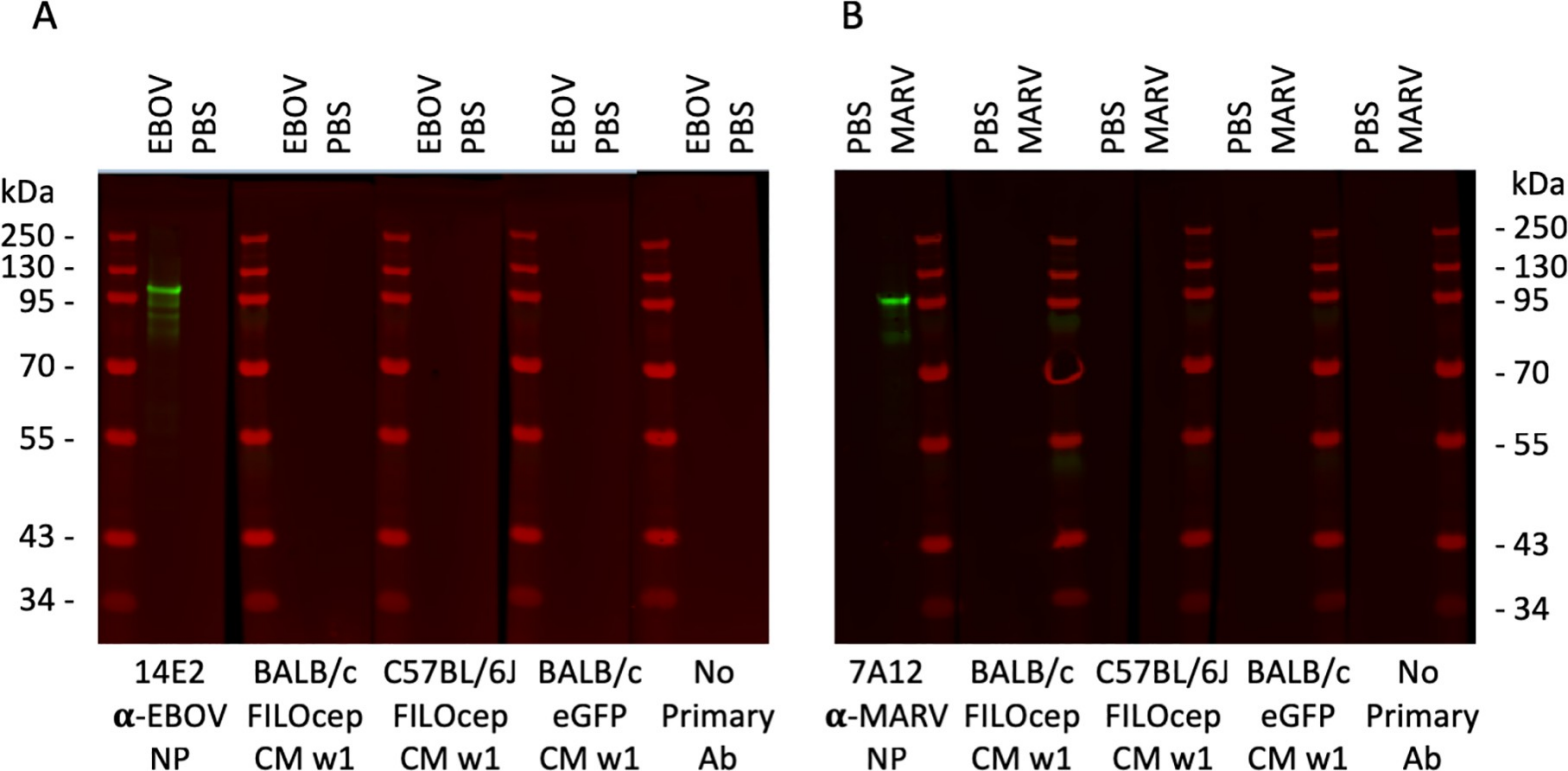
The FILOcep1&2 vaccine administration did not induce any filovirus-specific antibodies. Proteins in lysates of 8 μg of purified irradiated EBOV (A) or MARV (B) preparations were separated by SDS-PAGE and used to assess the induction of filovirus protein-specific antibodies induced by administration of the FILOcep1&2 vaccines in a Western blot. Sera from 1 week after the MVA.FILOcep1&2 boost of the BALB/c or C57BL/6J mice were combined for each group, diluted 1:1000 and tested against the Western blotted virus lysates or PBS as indicated. MAbs specific for the EBOV and MARV NPs served as positive controls, and no serum or sera from the same time isolated from mice vaccinated with the control eGPF vaccines were used as negative controls. Relative molecular mass markers are shown.

The challenge experiment followed the design in Table 1. Four mice were sacrificed on day 28 of the schedule to confirm induction of FILOcep1&2-specific T-cell responses in the vaccine recipients using the four most dominant peptide pools and some variability among animals in the relative frequencies of T-cells was noticed (Fig 5B). Following experimental challenge with Ebola and Marburg viruses of 8 animals per group, control animals started to lose their body mass and all died or were euthanized by day 7 post challenge with the exception of one MARV-challenged control mice, which regained mass and was still alive on day 28. In contrast, all mice which received the FILOcep1&2 vaccines kept gaining body mass and stayed alive till the end of the protocol (Fig 5C). Thus, the FILOcep1&2 vaccines protected against Ebola and Marburg viruses in both the BALB/c and C57BL/6J strains of mice carrying different H-2 molecules and presenting different peptides, and the vaccine-elicited T cells did so in the absence of challenge virus-specific antibodies (Fig 6).

## Discussion

In the present work, the ChAdOx1-MVA/FILOcep1&2 vaccines induced broadly specific, plurifunctional T-cell responses in mice and proved the concept that a pan-filovirus T-cell vaccine alone, in the absence of GP antibodies, can confer a 100% protection against experimental 1000 LD_50_ lethal challenges with filoviruses of two different genera and do so in the BALB/c and C57BL/6J strains of mice.

In our experience with chimeric T-cell immunogens similar to the FILOcep1&2 proteins delivered by DNA, recombinant simian adenoviruses and MVA, and administered to mice, NHPs and humans, induction of transgene product-specific antibodies was extremely rare [20–22]. Because the intracellularly expressed proteins in the absence of any naturally evolved folding are unstable and there is no surface GP included, no readily detectable filovirus-specific antibodies were induced. Therefore, we consider it highly unlikely that anti-FILOcep1&2 antibodies contributed the observed protection.

The strongest 15-mer peptides in the BALB/c mice were mapped in pools P3, P11 and P12. For a few epigraph variant pairs, the responses were similar, for other pairs, one variant was poorly or not recognized at all. This may reflect the differences that the bi-valent epigraph has to cover even for some of the most conserved protein regions; the coverage for variable regions must be worse. Two most immunodominant epitopes recognized by CD8^+^ T cells were narrowed down to the optimal length. The strongest epitope of the two, GS12, was conserved across the eight filoviruses. The other epitope AL10 happened to have a perfect match in FILOcep1 and FILOcep2 to the two challenge viruses EBOV and MARV, respectively, even though the EBOV variant yielded 5-fold lower specific T-cell frequencies and a great depth of recognition resulting from the bi-valent vaccine immunization was not achieved for this epitope. Thus, the coverage of epitopes in the filovirus species by the bi-valent vaccine will likely differ for each individual epitope and the protective effect against viruses will depend on the number of different epitopes recognized by the vaccine-elicited effector cells. Sometimes one protective invariant epitope may suffice, while recognition of multiple epitopes provides a better chance for virus control.

Previously, a protective role of T cells in immunity against EBOV in mice was suggested by studies in genetically modified mice [23] and by passive transfer of lymphocytes [24, 25] although in the one of the studies, a role for humoral immunity was also implicated [24]. A protection by T cells against several heterologous EBOV species was also reported by Hensley and colleagues [12].

There are a very few known HLA-restricted epitopes derived from filoviruses. The FILOcep1&2 regions span amino acids 131–420 in the nucleoprotein, 71–193 in matrix, and 540–854 and 952–1060 in the RNA polymerase, and of these the nucleoprotein has been the most studied. Searching the Immune Epitope Database (IEDB; https://www.iedb.org/) for known T-cell epitopes in filoviruses currently yields 10 well defined human CD8 T-cell epitopes, of which 7 are contained in the vaccine (Table 2). The number of HLA-restricted PTEs covered by the FILOcep1&2 vaccines can be estimated by shifting an 8-, 9- and 10-amino acid-long window across the 827-amino acid proteins, which gives 820 8-mer, 819 9-mer and 818 10-mer PTEs times two for the two FILOcep1&2 immunogens. Each of these PTEs then needs to be predicted for binding to major HLA alleles. This analysis would almost certainly yield more than sufficient number of human epitopes for all major HLAs to induce a broad response in every individual. Immunodominance will always be established narrowing down the response specificities. The protective potential of the FILOcep1&2 vaccine-elicited responses in humans can be only established by exposure of vaccinated individuals.

**Table 2:** Well defined human CD8^+^ T-cell epitopes in FILOcep1&2*.

Epitope	Virus of origin	Restriction
FLSFASLFL	EBOV	HLA-A02
RLMRTNFLI	RESTV	HLA-A02
AYQGDYKLF	EBOV	HLA-A23
FPQLSAIAL	EBOV	HLA-B35
FQQTNAMVTL	EBOV	HLA-A02
LHVVNYNGLL	EBOV	HLA-B15
VHAEQGLIQY	EBOV	HLA-A*30:02

*Immune Epitope Database (https://www.iedb.org/) as of Nov 2018.

Our next step is to determine whether or not the efficacy of T cells alone induced by the ChAdOx1-MVA/FILOcep1&2 vaccines translates to NHPs. If the mouse protection is replicated in NHP, identification of the correlates of protection in NHPs might greatly encourage testing the immunopotency of these vaccines in human volunteers. In the past, CD8^+^ T cells induced by HAdV-vectored vaccine conferred protection of NHPs against EBOV infection [26, 27]. The likely absence of an opportunity to demonstrate a human phase 3 efficacy may allow an alternative licensure pathway. A successful pan-filovirus vaccine would have multiple uses such as generation of vaccine stockpiles for containment of future outbreaks, elimination of the 2013 and 2018 outbreak remnants, elimination of virus reservoirs in survivors, provision of long-term protection in high risk populations including health workers and may even help saving highly endangered western gorillas.

## Materials and methods

### Synthetic genes for FILOcep1 and FILOcep2

Two DNA fragments carrying the two FILOcep1 and FILOcep2 ORFs were synthesized (Life Technologies) using humanized codons and were preceded by the consensus Kozak sequence to -5 nucleotides to maximize protein expression.

### Construction of the MVA.FILOcep1 and MVA.FILOcep2 vaccines

The parental non-replicating MVA originates directly from Professor Anton Mayr, passage 575 dated 14 December, 1983. The *FILOcep1*, *FILOcep2* or *eGFP* ORFs were cloned into transfer plasmid p856MVA-GFP-mH5 under control of the modified H5 promoter. Through homologous recombination, the expression cassettes were directed into the thymidine kinase locus on the MVA genome. Recombinant MVAs were made as described elsewhere. Briefly, chicken embryo fibroblast (CEF) cells grown in Dulbeco’s Modified Eagle’s Medium supplemented with 10% FBS, penicillin/streptomycin and glutamine (DMEM 10) were infected with parental MVA at MOI 1 and transfected using Superfectin (Qiagen) with 3 μg of p856MVA-GFP-TD-mH5.FILOcep1 or p856MVA-GFP-TD-mH5.FILOcep2 DNA. The cell lysate from this recombination was harvested and used to infect CEF. These cells were MoFlo-single cell sorted into 96-well plates and these were used to culture recombinant virus upon addition of fresh CEF. Those wells containing suitably infected cells were harvested and screened by PCR to confirm identity and test purity. Plaque picking was performed until the culture was free of parental virus, as determined by PCR. The virus was then bulk-prepared and purified on a 36% sucrose cushion, titred and stored at –80 ^o^C until use.

### Construction of the ChAdOx1.FILOcep1 and ChAdOx1.FILOcep2 vaccines

The ChAdOx1 vaccine vector is derived from ChAdV isolate Y25 of group E adenoviruses, and pre-existing antibodies to group E are rare in human populations. Its genome modifications include removal of the E1, E3 and a substitution of simian region E4 with the HAdV-5 E4 *orf4* and *orf6/7* genes. For the generation of recombinant ChAdOx1s, the *FILOcep1* and *FILOcep2* ORFs were subcloned under the control of the human cytomegalovirus immediate early promoter into plasmid pENTR4_Mono and inserted at the E1 locus of the ChAdOx1 genome by GalK recombineering. Recombinant ChAdOx1 vaccines were rescued by transfection of HEK293A T-Rex cells (Invitrogen/ThermoFisher Scientific) using linearized plasmid. The presence of the transgene and absence of contaminating empty parental adenovirus were confirmed by PCR. The virus was titred to determine infectious units (IU) per ml, assayed by spectrophotometry to quantify the number of virus particles per ml and stored at –80 ^o^C until use.

### Mice, immunizations and preparation of splenocytes

Six-week-old female BALB/c or C57BL/6 mice were purchased from Envigo (UK) and housed at the Functional Genomics Facility, University of Oxford. Mice were immunized intramuscularly under general anesthesia either with varying amounts of rChAdOx1s and rMVAs. On the day of sacrifice, spleens were collected and cells isolated by pressing organs individually through a 70-μm nylon mesh of a sterile cell strainer (Fisher Scientific) using a 5-ml syringe rubber plunger. Following the removal of red blood cells (RBC) with RBC Lysing Buffer Hybri-Max (Sigma), splenocytes were washed and resuspended in R10 (RPMI 1640 supplemented with 10% FCS, penicillin/streptomycin and β-mercaptoethanol) for ELISPOT and intracellular cytokine staining (ICS) assays.

### Peptides and peptide pools

All peptides were at least 90% pure by mass spectrometry (Ana Spec, San Jose, CA, USA and Synpeptide Co Ltd, Shanghai, China), were dissolved in DMSO (Sigma-Aldrich) to yield a stock of 10 mg/ml, and stored at –80°C. Three hundred and ninety FILOcep1&2-derived peptides 15-mer overlapping by 11 amino acids were divided into 12 pools P1-P12 of 34 to 47 individual peptides in a way that variant peptides were always present in the same pool for use in ICS and ELISPOT assays. 17 pairs of stimulatory ‘BALB/c’ peptides were employed as specified in each figure. The peptides were used at a final concentration of 1.5 μg/ml each.

### The IFN-γ ELISPOT assay

The ELISPOT assay was performed using the Mouse IFN-γ ELISpot kit (Mabtech, Stockholm, Sweden) or FluoroSpot kits (Mabtech and Cellular Technology Limited, Cleveland, OH, USA) according to the manufacturer’s instructions. For the former, immune splenocytes were collected and tested separately from individual mice. Peptides were used at 1.5 μg/ml each and splenocytes at 5 × 10^4^ cells/well were added to 96-well high protein binding Immobilon-P membrane plates (Millipore) that had been precoated with 5 μg/ml anti-IFN-γ mAb AN18 (Mabtech,). The plates were incubated at 37°C in 5% CO_2_ for 18 hours and washed with PBS before the addition of 1 μg/ml biotinylated anti-IFN-γ mAb (Mabtech) at room temperature for 2 hours. The plates were then washed with PBS, incubated with 1 μg/ml streptavidin-conjugated alkaline phosphatase (Mabtech) at room temperature for 1 hour, washed with PBS, and individual cytokine-producing units were detected as dark spots after a 10-minute reaction with 5-bromo-4-chloro-3-idolyl phosphate and nitro blue tetrazolium using an alkaline phosphatase-conjugate substrate (Bio-Rad, Richmond, CA, USA). Spot-forming units were counted using the AID ELISpot Reader System (Autoimmun Diagnostika). The frequencies of responding cells were expressed as a number of spot-forming units/10^6^ splenocytes.

### Intracellular cytokine staining (ICS) assay

Splenocytes or PBMCs isolated from whole blood were stimulated with peptide at 2 μg/ml, ionomycin and phorbol myristate acetate (PMA) at 2.0 μg/ml and 0.5 μg/ml, respectively, or tissue culture media with 1% DMSO as a negative control. The cultures were supplemented with anti-CD107a PE-conjugated mAb (eBioscience). The cells were incubated at 37 ^o^C, 5% CO_2_ for 2 hours prior to the addition of Brefeldin A and monensin (BD Biosciences) and then left in culture overnight. The cells were centrifuged briefly, washed in PBS plus 5% BSA (Sigma-Aldrich) and the pellet re-suspended in 40 μl of CD16/32 with LIVE/DEAD fixable aqua stain (Molecular Probes, Invitrogen). Cells were washed, a mastermix of anti-membrane marker mAbs was prepared containing CD4 APC/Cy7 (Biolegend), CD3 PerCP-eFluor710 and CD8a eFluor 450 (both from eBioscience) and 40 μl added to each tube. The cells were incubated at 4 ^o^C for 30 min and then permeabilized using Fix/Perm solution (Becton-Dickinson) for 20 min at 4 ^o^C. The cells were washed with Perm Wash buffer (Becton Dickinson) and a mastermix of anti-intracellular molecule mAbs was prepared containing IFN-γ PE-Cy7, IL-2 APC and TNF-α FITC (all from eBioscience). The cells were incubated at 4 ^o^C for 30 min, washed and resuspended in Perm Wash buffer prior to running on an LSRII flow cytometer (Becton-Dickinson).

### Ebola and Marburg virus challenge studies

Groups of eight 6- to 7-week-old BALB/c or C57BL/6J female mice (Charles River) were vaccinated intramuscularly under general anesthesia with 1x10^8^ IU total of rChAdOx1s followed by 1x10^7^ PFU total of MVAs. At day 35 of the protocol or day 24 post-vaccination (Table 1), all the mice received a challenge dose of 1000x the 50% lethal dose (LD_50_) of either mouse-adapted EBOV or mouse-adapted MARV in 200 μl of DMEM (pH 7.4) by intraperitoneal injection. All animals were monitored daily for signs of disease, survival and body-mass change for 14 days followed by additional 14 days monitoring of survival.

### Viral RNA detection by RT-qPCR

FILOcep1&2- and control eGFP-vaccinated mice were challenged with either mouse-adapted EBOV or MARV. Blood and tissues (liver, spleen, kidney and lungs) from 4 mice per vaccinated group were collected upon euthanasia at day 3 and 5 post-infection to determine viral RNA levels. RNA of mouse blood and tissues were extracted using QIAamp viral RNA minikit (Qiagen) and the RNeasy mini Kit (Qiagen) according to the manufacturer's instructions. Viral RNA levels were quantified by reverse transcription quantitative PCR (RT-qPCR) targeting viral polymerase gene and using the Light Cycler 480 thermal cycler (Roche, Germany). The primers and probes are shown in Table 3. Cycling conditions were as follows: 63°C for 3 min and 95°C for 30 sec, followed by 45 cycles of 95°C for 15 sec and 60°C for 30 sec.

**Table 3:** Primers and probes for determination of virus load.

EBOV	Primer-Forward	5’-CAGCCAGCAATTTCTTCCAT-3’,
	Primer- Reverse	5’-TTTCGGTTGCTGTTTCTGTG-3’
	Probe	5’-[FAM]-ATCATTGGCGTACTGGAGGAGCAG-[TAMRA]
MARV	Primer 1-Forward	5′-GCAAAAGCATTCCCTAGTAACATGA-3′
	Primer 1- Reverse	5′-CACCCCTCACTATRGCGTTYTC-3′
	Primer 2-Forward	5′-GCGAAGGCATTCCCTAGTAATATGA-3′
	Primer 2- Reverse	5′-CACCTCTTACTATGGCATTCTC-3′
	Probe	5′-56-FAM/TGGCACCAY/ZEN/AATTCAGCAAGCATAGG/ 3IABkFQ-3

### Western blot analysis of mouse sera

EBOV and MARV concentrates were prepared from virus-infected Vero cell culture supernatants by unlracentrifugation and inactivation by γ-irradiation. Viruses were lysed in a loading buffer and an equivalent of 8 μg of virus or PBS were separated on 15% SDS-polyacrylamide gel and transferred onto a nylon filter (Amersham International), and the filters were blocked and incubated with mAbs 14E2 (EBOV NP), 7A12 (MARV NP) or combined mouse sera from each animal group diluted 1:1000. Bound antibodies were detected using horse radish peroxidase (HRP)-conjugated protein A (Amersham International) followed by enhanced chemiluminiscence (ECL; Amersham International).

### Statistical analysis

Statistical analyses were performed using Graph Pad Prism version 7. Responses were assumed to be non-Gaussian in distribution, thus non-parametric tests were used throughout and medians (range) are shown. Multiple comparisons were performed using the Kruskal-Wallis test. Groups with the same *in vitro* restimulations were compared using two-tailed Mann-Whitney U tests. Two-tailed *P* values were used and *P* values of less than 0.05 were considered statistically significant.

### Ethics statement

Chicken embryo fibroblasts were prepared at Poultry Health Services Ltd, Huntingdon, UK and, in the United Kingdom, there is no need for Ethics permission for killing 7-day-old chicken embryos.

All mouse procedures and care in Oxford were approved by the local Clinical Medicine Ethical Review Committee, University of Oxford and conformed strictly to the United Kingdom Home Office Guidelines under the Animals (Scientific Procedures) Act 1986. Experiments were conducted under Project License 30/3387 held by T.H.

All the animal challenge experiments were performed in the biological safety level 4 (BSL-4) facility at the Canadian Science Centre for Human and Animal Health (CSCHAH) in Winnipeg, Canada. All mouse procedures and care at the Canadian Science Center for Human and Animal Health (CSCHAH) were approved by the local Animal Care Committee and conformed strictly to the Canadian Council on Animal Care (CCAC). Experiments were conducted under Animal Use Document H17-007 held by X.Q.

## Supporting information

S1 Fig Mapping of stimulatory 15-mer peptides in the BALB/c and C57BL/6J strains of mice. Groups of mice were immunized using the ChAdOx1.FILOcep1 + ChAdOx1.FILOcep2 prime and MVA.FILOcep1 + MVA.FILOcep2 boost regimen and the immune splenocytes were tested using the IFN-y ELISPOT assay against 390 individual peptides corresponding to the FILOcep1&2 immunogens.
(PDF)

S1 Data (PDF)
