# Supplementary material for: Complete protection of the BALB/c and C57BL/6J mice against Ebola and Marburg virus lethal challenges by pan-filovirus T-cell epigraph vaccine
Source: PLoS Pathog. 2019 Feb 28;15(2):e1007564. doi: 10.1371/journal.ppat.1007564 (PMC6394903; doi:10.1371/journal.ppat.1007564)
Supplement: S1 Fig — Groups of mice were immunized using the ChAdOx1.FILOcep1 + ChAdOx1.FILOcep2 prime and MVA.FILOcep1 + MVA.FILOcep2 boost regimen and the immune splenocytes were tested using the IFN-y ELISPOT assay against 390 individual peptides corresponding to the FILOcep1&2 immunogens. (PDF) [file ppat.1007564.s001.pdf]

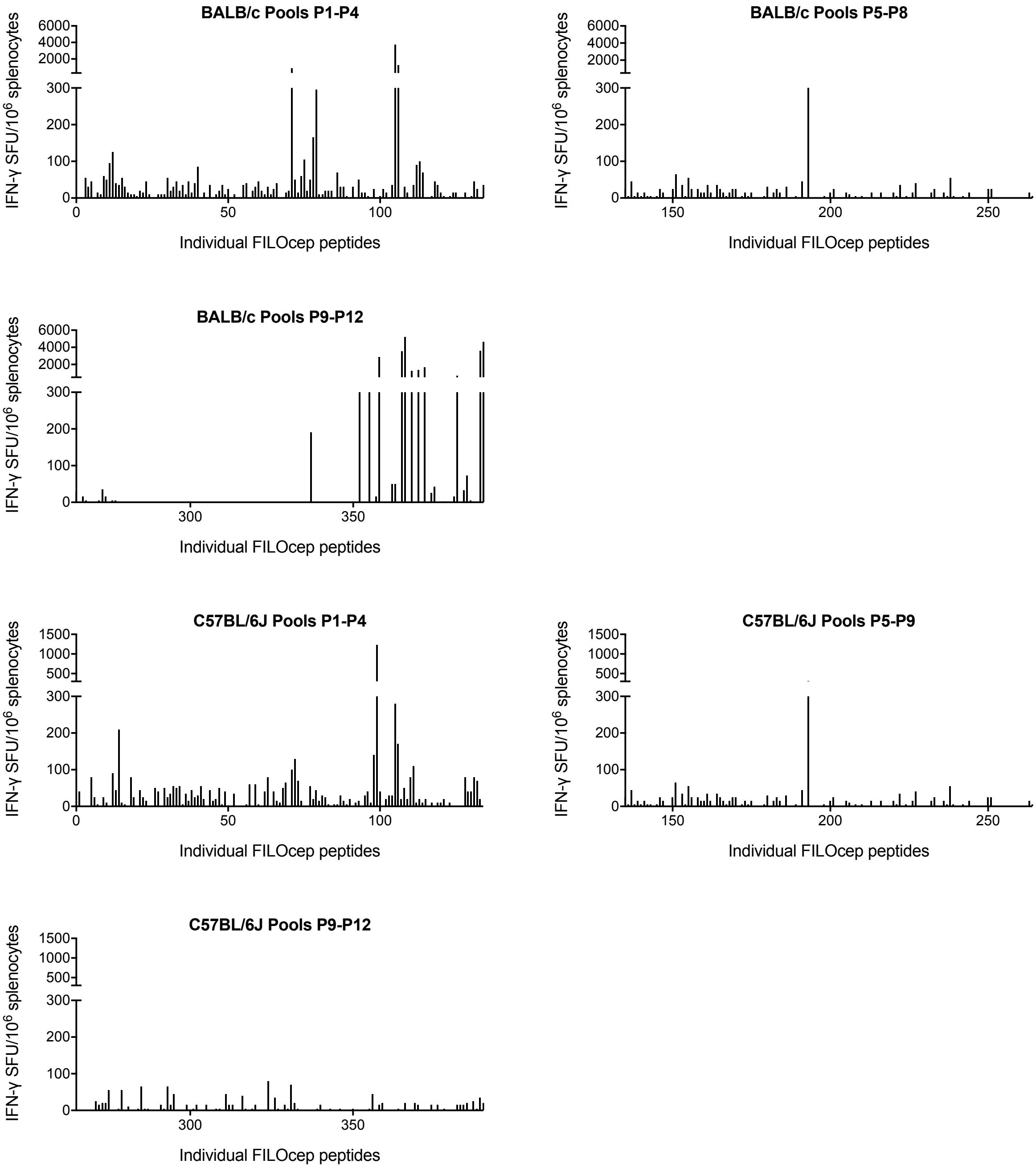

S1 Fig. Mapping of stimulatory 15-mer peptides in the BALB/c and C57BL/6J strains of mice. Groups of mice were immunized using the ChAdOx1.FILOcep1+ChAdOx1.FILOcep2 prime and MVA.FILOcep1+MVA.FILOcep2 boost regimen and the immune splenocytes were tested using the IFN-γ ELISPOT assay against 390 individual peptides corresponding to the FILOcep1&2 immunogens.
