# Supplementary material for: Complete protection of the BALB/c and C57BL/6J mice against Ebola and Marburg virus lethal challenges by pan-filovirus T-cell epigraph vaccine
Source: PLoS Pathog. 2019 Feb 28;15(2):e1007564. doi: 10.1371/journal.ppat.1007564 (PMC6394903; doi:10.1371/journal.ppat.1007564)
Supplement: S1 Data — (PDF) [file ppat.1007564.s002.pdf]

Fig 2A-1

Table format: Grouped		Group A $1x10^6$ IU dose			Group B $1x10^7$ IU dose			Group C $1x10^8$ IU dose			Group D $5x10^8$ IU dose		
		A:Y1	A:Y2	A:Y3	B:Y1	B:Y2	B:Y3	C:Y1	C:Y2	C:Y3	D:Y1	D:Y2	D:Y3
1	P1	0	17.5	10	0.0	55.0	10	125.0	295.0	14	260	350	275.0
2	P2	20	45.0	70	0.0	305.0	0	27.5	2.5	5	35	210	0.0
3	P3	0	37.5	60	0.0	85.0	30	395.0	295.0	260	460	210	125.0
4	P4	30	7.5	40	0.0	65.0	0	95.0	15.0	5	20	40	37.5
5	P5	0	7.5	40	0.0	35.0	0	55.0	85.0	120	20	30	25.0
6	P6	10	0.0	5	7.5	35.0	60	55.0	45.0	15	230	320	55.0
7	P7	0	17.5	0	0.0	65.0	20	245.0	185.0	30	400	270	355.0
8	P8	0	47.5	15	0.0	7.5	0	105.0	75.0	5	80	160	45.0
9	P9	0	0.0	20	0.0	45.0	10	7.5	35.0	0	20	40	0.0
10	P10	0	7.5	15	0.0	15.0	60	185.0	75.0	0	360	90	85.0
11	P11	0	0.0	0	35.0	35.0	20	0.0	42.5	50	50	120	105.0
12	P12	20	135.0	150	300.0	295.0	380	1765.0	1935.0	1070	2070	2880	2365.0
13	Title												

Fig 2A-2

Table format: Grouped		Group A 1x10^6 IU dose			Group B 1x10^7 IU dose			Group C 1x10^8 IU dose			Group D 5x10^8 IU dose		
		A:Y1	A:Y2	A:Y3	B:Y1	B:Y2	B:Y3	C:Y1	C:Y2	C:Y3	D:Y1	D:Y2	D:Y3
1	P1	55	25.0	0	2.5	27.5	20	10	0	90	5	10	35.0
2	P2	5	15.0	10	12.5	117.5	10	40	0	10	0	80	2.5
3	P3	50	2.5	70	175.0	175.0	260	2180	820	1940	1390	1010	1155.0
4	P4	15	22.5	0	5.0	7.5	50	60	0	30	25	20	15.0
5	P5	0	5.0	10	35.0	0.0	5	30	0	30	0	0	12.5
6	P6	15	12.5	10	22.5	0.0	5	70	10	50	0	60	12.5
7	P7	0	0.0	10	25.0	0.0	5	60	0	30	45	10	2.5
8	P8	55	2.5	0	35.0	7.5	10	50	30	90	0	30	95.0
9	P9	25	35.0	10	35.0	7.5	70	70	0	60	25	0	32.5
10	P10	15	15.0	10	15.0	17.5	45	130	0	240	80	110	72.5
11	P11	0	0.0	10	25.0	7.5	0	40	0	90	30	50	15.0
12	P12	110	12.5	60	65.0	145.0	40	74	570	690	480	750	515.0

Fig 2A-3

		Group A $1x10^6$ IU dose			Group B $1x10^7$ IU dose			Group C $1x10^8$ IU dose			Group D $5x10^8$ IU dose		
		A:Y1	A:Y2	A:Y3	B:Y1	B:Y2	B:Y3	C:Y1	C:Y2	C:Y3	D:Y1	D:Y2	D:Y3
1	P1	0.0	10	25.0	0.0	160	65.0	395	265	250	200	265	240
2	P2	55.0	30	7.5	0.0	105	17.5	5	65	0	0	45	25
3	P3	27.5	190	125.0	305.0	550	245.0	805	705	1180	820	1485	1370
4	P4	7.5	30	55.0	32.5	25	45.0	85	55	20	40	35	5
5	P5	0.0	0	0.0	0.0	15	15.0	5	35	40	90	15	120
6	P6	47.5	10	47.5	12.5	10	17.5	5	15	10	100	65	140
7	P7	0.0	40	7.5	5.0	50	27.5	85	85	180	40	255	160
8	P8	0.0	20	17.5	0.0	60	7.5	175	95	160	50	0	140
9	P9	0.0	10	17.5	12.5	20	0.0	65	45	0	0	85	50
10	P10	7.5	0	47.5	2.5	20	35.0	55	165	50	60	185	110
11	P11	0.0	10	0.0	0.0	5	15.0	95	55	100	80	25	90
12	P12	215.0	130	275.0	395.0	550	305.0	2035	1935	3150	1610	2635	1900

Fig 2A-4

Table format: Grouped		Group A ChAdOx1.EBOVcep1			Group B ChAdOx1.EBOVcep2			Group C ChAdOx1.EBOVcep1+2		
		A:Y1	A:Y2	A:Y3	B:Y1	B:Y2	B:Y3	C:Y1	C:Y2	C:Y3
1	P1	125.0	295.0	14	10	0	90	395	265	250
2	P2	27.5	2.5	5	40	0	10	5	65	0
3	P3	395.0	295.0	260	2180	820	1940	805	705	1180
4	P4	95.0	15.0	5	60	0	30	85	55	20
5	P5	55.0	85.0	120	30	0	30	5	35	40
6	P6	55.0	45.0	15	70	10	50	5	15	10
7	P7	245.0	185.0	30	60	0	30	85	85	180
8	P8	105.0	75.0	5	50	30	90	175	95	160
9	P9	7.5	35.0	0	70	0	60	65	45	0
10	P10	185.0	75.0	0	130	0	240	55	165	50
11	P11	0.0	42.5	50	40	0	90	95	55	100
12	P12	1765.0	1935.0	1070	74	570	690	2035	1935	3150

Fig 2B-5

Table format: Grouped		Group A 1X10^5 PFU dose			Group B 1x10^6 PFU dose			Group C 5x10^6 PFU dose			Group D 1x10^7 PFU dose		
		A:Y1	A:Y2	A:Y3	B:Y1	B:Y2	B:Y3	C:Y1	C:Y2	C:Y3	D:Y1	D:Y2	D:Y3
1	P1	30.0	17.5	30.0	0.0	155.0	60	175.0	35.0	130	10.0	30	5.0
2	P2	10.0	35.0	30.0	0.0	395.0	30	137.5	132.5	45	35.0	60	0.0
3	P3	50.0	17.5	50.0	0.0	325.0	0	235.0	85.0	220	30.0	150	45.0
4	P4	20.0	17.5	50.0	0.0	235.0	20	195.0	85.0	15	30.0	100	57.5
5	P5	0.0	0.0	60.0	0.0	85.0	160	125.0	25.0	90	50.0	40	35.0
6	P6	40.0	0.0	35.0	17.5	145.0	90	295.0	45.0	45	130.0	10	15.0
7	P7	20.0	0.0	0.0	0.0	145.0	100	175.0	25.0	230	60.0	30	35.0
8	P8	0.0	17.5	0.0	0.0	107.5	30	175.0	85.0	85	50.0	60	35.0
9	P9	0.0	0.0	10.0	0.0	105.0	80	67.5	35.0	0	70.0	0	0.0
10	P10	0.0	17.5	0.0	0.0	135.0	80	325.0	105.0	0	40.0	60	75.0
11	P11	0.0	0.0	0.0	45.0	215.0	200	0.0	32.5	220	70.0	130	35.0
12	P12	50.0	55.0	20.0	85.0	245.0	30	255.0	85.0	200	100.0	90	35.0

Fig 2B-6

Table format: Grouped		Group A 1X10^5 PFU dose			Group B 1x10^6 PFU dose			Group C 5x10^6 PFU dose			Group D 1x10^7 PFU dose		
		A:Y1	A:Y2	A:Y3	B:Y1	B:Y2	B:Y3	C:Y1	C:Y2	C:Y3	D:Y1	D:Y2	D:Y3
1	P1	0	65.0	0	12.5	27.5	110	120	0	50	0	10	45.0
2	P2	15	35.0	10	42.5	47.5	80	70	0	70	0	10	52.5
3	P3	120	132.5	150	205.0	185.0	370	210	432	220	80	160	255.0
4	P4	35	22.5	0	35.0	0.0	140	0	0	50	0	0	125.0
5	P5	0	55.0	50	85.0	0.0	25	70	0	0	0	20	22.5
6	P6	65	22.5	20	32.5	0.0	15	70	10	70	0	20	42.5
7	P7	0	0.0	30	35.0	0.0	55	90	0	20	25	10	2.5
8	P8	5	2.5	10	45.0	47.5	90	80	50	60	0	10	5.0
9	P9	0	45.0	10	65.0	7.5	70	10	30	10	0	50	12.5
10	P10	5	5.0	20	45.0	0.0	35	60	0	60	30	60	12.5
11	P11	0	0.0	0	45.0	7.5	0	110	0	80	20	60	45.0
12	P12	0	0.0	110	105.0	15.0	160	160	180	60	70	20	95.0

Fig 2B-7

Table format: Grouped		Group A 1X10^5 PFU dose			Group B 1x10^6 PFU dose			Group C 5x10^6 PFU dose			Group D 1x10^7 PFU dose		
		A:Y1	A:Y2	A:Y3	B:Y1	B:Y2	B:Y3	C:Y1	C:Y2	C:Y3	D:Y1	D:Y2	D:Y3
1	P1	0.0	10	5.0	0.0	10	55.0	125	95	60	150	85.0	150
2	P2	0.0	0	27.5	0.0	55	27.5	105	105	10	0	52.5	45
3	P3	0.0	70	115.0	25.0	170	215.0	165	155	160	220	345.0	170
4	P4	0.0	0	95.0	0.0	45	65.0	65	95	40	120	115.0	45
5	P5	0.0	0	0.0	0.0	15	75.0	55	0	0	90	65.0	50
6	P6	0.0	0	17.5	12.5	10	47.5	55	15	70	110	85.0	80
7	P7	0.0	0	0.0	0.0	0	37.5	75	75	70	70	55.0	0
8	P8	0.0	0	27.5	0.0	0	0.0	25	115	40	40	0.0	40
9	P9	0.0	0	0.0	22.5	0	0.0	65	65	20	0	75.0	60
10	P10	0.0	0	27.5	0.0	40	35.0	65	65	60	190	175.0	0
11	P11	0.0	0	0.0	0.0	55	75.0	55	35	40	160	75.0	40
12	P12	0.0	0	15.0	5.0	10	105.0	105	165	60	150	125.0	90

Fig 2B-8

Table format: Grouped		Group A 1X10^5 PFU dose			Group B 1x10^6 PFU dose			Group C 5x10^6 PFU dose			Group D 1x10^7 PFU dose		
		A:Y1	A:Y2	A:Y3	B:Y1	B:Y2	B:Y3	C:Y1	C:Y2	C:Y3	D:Y1	D:Y2	D:Y3
1	P1	0.0	10	5.0	0.0	10	55.0	125	95	60	150	85.0	150
2	P2	0.0	0	27.5	0.0	55	27.5	105	105	10	0	52.5	45
3	P3	0.0	70	115.0	25.0	170	215.0	165	155	160	220	345.0	170
4	P4	0.0	0	95.0	0.0	45	65.0	65	95	40	120	115.0	45
5	P5	0.0	0	0.0	0.0	15	75.0	55	0	0	90	65.0	50
6	P6	0.0	0	17.5	12.5	10	47.5	55	15	70	110	85.0	80
7	P7	0.0	0	0.0	0.0	0	37.5	75	75	70	70	55.0	0
8	P8	0.0	0	27.5	0.0	0	0.0	25	115	40	40	0.0	40
9	P9	0.0	0	0.0	22.5	0	0.0	65	65	20	0	75.0	60
10	P10	0.0	0	27.5	0.0	40	35.0	65	65	60	190	175.0	0
11	P11	0.0	0	0.0	0.0	55	75.0	55	35	40	160	75.0	40
12	P12	0.0	0	15.0	5.0	10	105.0	105	165	60	150	125.0	90

Fig 3A-1

Table format: Grouped		Group A ChAdOx1.FILOcep			Group B MVA.gfp.FILOcep			Group C ChAdOx1.EBOVcep-prime MVA.gfp.FILO		
		A:Y1	A:Y2	A:Y3	B:Y1	B:Y2	B:Y3	C:Y1	C:Y2	C:Y3
1	P1	395	265	250	150	85.0	150	955	660	415
2	P2	5	65	0	0	52.5	45	55	70	15
3	P3	805	705	1180	220	345.0	170	4515	4720	5015
4	P4	85	55	20	120	115.0	45	205	80	85
5	P5	5	35	40	90	65.0	50	55	50	25
6	P6	5	15	10	110	85.0	80	115	30	25
7	P7	85	85	180	70	55.0	0	325	30	1105
8	P8	175	95	160	40	0.0	40	205	40	105
9	P9	65	45	0	0	75.0	60	55	130	165
10	P10	55	165	50	190	175.0	0	165	140	225
11	P11	95	55	100	160	75.0	40	345	280	235
12	P12	2035	1935	3150	150	125.0	90	6085	5760	4845
13	Title									

Fig 3B-2

Table format: Grouped		Group A C1C2-Left & M1M2-Right			Group B C1M1-Left & C2M2-Right			Group C C1C2M1M2-Left & Right		
		A:Y1	A:Y2	A:Y3	B:Y1	B:Y2	B:Y3	C:Y1	C:Y2	C:Y3
1	P1	405	210	385	135	120	105	125	75	90
2	P2	5	0	0	15	10	0	85	5	40
3	P3	1285	710	1075	755	730	415	595	725	1090
4	P4	495	60	45	35	50	45	115	75	0
5	P5	55	50	75	15	100	0	5	0	50
6	P6	125	20	0	55	50	125	45	25	0
7	P7	365	120	115	85	120	45	15	15	50
8	P8	325	50	55	155	60	15	145	15	30
9	P9	105	0	15	65	50	0	85	25	10
10	P10	115	40	195	35	110	15	135	55	0
11	P11	225	300	235	55	50	135	85	125	40
12	P12	1435	1750	2225	905	1560	1165	1315	935	880
13	Title									

Fig 3C-3

new sheet rmat: Grouped		Group A				Group B				Group C			
		-- C1C2M1M2				C1C2M1M2 --				C1C2-M1M2			
		A:Y1	A:Y2	A:Y3	A:Y4	B:Y1	B:Y2	B:Y3	B:Y4	C:Y1	C:Y2	C:Y3	C:Y4
1	P1	230	110	350	150	50	110	110	120	470	350	170	280
2	P2	10	60	70	20	40	50	50	80	120	40	70	10
3	P3	720	610	810	370	460	730	660	690	3230	2490	2620	4060
4	P4	140	120	60	130	100	30	50	60	260	100	40	40
5	P5	60	60	90	80	40	20	70	30	120	50	30	60
6	P6	30	60	60	10	100	50	30	30	120	120	50	30
7	P7	90	170	110	110	80	140	40	70	110	370	120	70
8	P8	170	50	80	70	50	30	30	90	540	300	130	80
9	P9	100	50	50	50	0	60	40	90	180	70	110	140
10	P10	110	90	100	40	30	100	50	50	140	160	150	90
11	P11	170	140	260	90	90	90	80	40	380	380	50	120
12	P12	2210	1590	1810	1130	1250	1120	1270	990	4440	3260	3220	4490
13	Title												

Fig 3E-4

Table format: Grouped		Group A				Group B				Group C				Group D			
		IFN-γ				TNF-α				IL-2				CD107a			
		A:Y1	A:Y2	A:Y3	A:Y4	B:Y1	B:Y2	B:Y3	B:Y4	C:Y1	C:Y2	C:Y3	C:Y4	D:Y1	D:Y2	D:Y3	D:Y4
1	71/105	6.80	4.70	1.64	0.55	3.54	2.61	1.53	0.48	0.10	0.05	0.06	0.05	6.71	4.53	0.22	0.16
2	358/383	0.25	2.78	0.01	0.94	0.00	1.47	0.39	0.00	0.03	0.04	0.00	0.05	0.00	2.71	0.00	0.00
3	366/390	4.88	3.57	0.49	0.34	1.65	1.55	0.17	0.15	0.00	0.01	0.00	0.03	6.11	4.09	0.00	0.00
4	347/372	0.35	2.52	0.00	0.00	0.00	1.03	0.00	0.00	0.13	0.36	0.01	3.00e-003	0.33	2.60	0.00	0.00
5	Title																

Fig 3E-5

Table format: Grouped	Group A IFN-γ				Group B TNF-α				Group C IL-2				Group D CD107a			
	A:Y1	A:Y2	A:Y3	A:Y4	B:Y1	B:Y2	B:Y3	B:Y4	C:Y1	C:Y2	C:Y3	C:Y4	D:Y1	D:Y2	D:Y3	D:Y4
1 71/105	0.16	0.80	0.12	0.35	0.010	0.000	0.000	0.000	0.00	0.00	0.67	1.42	0	0	0	0
2 358/383	0.04	0.36	0.00	0.44	0.000	0.000	0.000	0.000	0.04	0.00	0.00	0.92	0	0	0	0
3 366/390	0.00	0.00	0.00	0.55	0.029	0.008	0.000	0.000	0.00	0.00	0.00	0.86	0	0	0	0
4 347/372	0.00	0.00	0.00	0.00	0.034	0.021	0.000	0.005	0.00	0.00	0.00	0.00	0	0	0	0
5 Title																

Fig 4A-1

Table format: Grouped		Group A Control				Group B FILOcep1&2			
		A:Y1	A:Y2	A:Y3	A:Y4	B:Y1	B:Y2	B:Y3	B:Y4
1	Pool 3	80	70	20	120	3290	1910	3210	3060
2	Pool 12	200	90	10	40	2730	3970	4710	6430
3	No peptide	190	40	0	130	110	40	120	90

Fig 4A-2

Table format: Grouped		Group A				Group B			
		Control				FILOcep1&2			
		A:Y1	A:Y2	A:Y3	A:Y4	B:Y1	B:Y2	B:Y3	B:Y4
1	Pool 3	80	20	100	30	1310	1110	1620	1730
all data tables	[illegible]	30	60	40	40	1440	2470	2580	4670
3	No peptide	150	60	40	140	40	50	40	60
4	Title								

Fig 4B-3

		X	Group A								Group B							
		DPI	Control								FiloCep vaccinated							
		X	A:Y1	A:Y2	A:Y3	A:Y4	A:Y5	A:Y6	A:Y7	A:Y8	B:Y1	B:Y2	B:Y3	B:Y4	B:Y5	B:Y6	B:Y7	B:Y8
1	Title	1	20.1	18.2	20.2	19.5	20.3	21.3	18.3	21.6	20.2	18.9	20.8	20.0	21.8	19.9	20.4	19.6
2	Title	2	20.6	18.3	20.4	19.9	20.6	21.5	18.8	22.2	20.8	19.2	21.1	20.8	21.7	20.8	21.3	20.2
3	Title	3	19.8	17.5	19.4	18.7	19.3	20.0	17.5	20.5	20.6	18.9	20.9	20.2	21.6	20.5	20.9	19.9
4	Title	4	19.2	16.3	18.7	16.5	17.1	17.8		18.6	20.4	18.5	20.7	20.2	21.4	20.3	20.8	19.9
5	Title	5	18.7	15.5			16.8				20.5	18.5	20.5	19.9	21.6	20.4	21.1	19.8
6	Title	6	17.1								20.3	18.4	20.4	19.9	21.1	19.5	20.8	19.4
7	Title	7									20.6	19.2	20.7	20.1	21.5	19.9	21.2	20.2
8	Title	8									20.3	18.9	20.3	20.1	21.1	19.8	21.0	19.8
9	Title	9									20.8	19.5	20.2	21.1	22.3	20.6	22.0	20.9
10	Title	10									20.6	19.4	20.5	20.9	22.0	20.5	21.9	20.6
11	Title	11									20.3	19.7	20.9	20.9	22.0	20.3	21.5	20.5
12	Title	12									20.1	19.4	20.6	20.6	21.8	20.2	22.5	20.1
13	Title	13									20.8	19.5	21.7	22.0	22.4	21.0	22.3	20.7
14	Title	14									20.1	20.0	21.0	21.3	21.4	20.1	21.2	19.6
15	Title	15									20.7	20.6	21.5	21.7	21.4	20.0	21.3	19.7
16	Title	16									20.3	20.2	20.9	21.3	21.5	19.9	20.9	19.6

Fig 4B-4

Table format: Survival		X	Group A	Group B
		DPI	Control	FiloCep vaccinat
		X	Y	Y
1	Title	4	1	
2	Title	4	1	
3	Title	5	1	
4	Title	5	1	
5	Title	5	1	
6	Title	6	1	
7	Title	6	1	
8	Title	7	1	
9	Title	28		0
10	Title	28		0
11	Title	28		0
12	Title	28		0
13	Title	28		0
14	Title	28		0
15	Title	28		0
16	Title	28		0

Fig 4B-5

		X	Group A								Group B							
		DPI	Control								FiloCep vaccinated							
		X	A:Y1	A:Y2	A:Y3	A:Y4	A:Y5	A:Y6	A:Y7	A:Y8	B:Y1	B:Y2	B:Y3	B:Y4	B:Y5	B:Y6	B:Y7	B:Y8
1	Title	1	21.2	19.3	20.1	19.8	19.6	20.0	20.4	20.6	19.9	19.8	22.3	19.2	20.7	21.2	18.6	20.2
2	Title	2	21.4	20.0	20.9	20.0	19.8	20.2	20.7	20.9	19.8	20.0	22.2	19.2	20.9	21.0	18.9	20.2
3	Title	3	20.2	18.3	19.4	19.2	19.0	19.0	19.2	19.4	19.6	19.8	22.3	18.8	20.4	21.2	19.1	19.6
4	Title	4	20.3	18.0	19.6	17.8	18.3	18.6	19.1	19.0	18.5	18.3	20.8	18.0	19.6	21.2	18.9	20.1
5	Title	5	18.3	16.3	17.2	16.3	16.6	17.0	17.0	17.5	20.2	19.9	22.2	19.5	19.9	20.8	18.8	19.4
6	Title	6		16.5			16.2		17.0		19.7	19.7	21.8	19.1	19.9	20.9	19.5	20.1
7	Title	7									19.3	19.5	21.5	19.1	19.9	21.1	19.3	20.4
8	Title	8									19.4	19.7	21.2	19.0	19.8	20.5	18.8	20.2
9	Title	9									20.3	20.3	22.1	19.3	20.8	20.7	19.2	20.4
10	Title	10									21.0	19.8	21.6	19.4	19.9	20.9	19.6	20.4
11	Title	11									20.7	20.7	22.5	19.6	19.9	21.2	19.8	21.0
12	Title	12									20.6	20.1	22.8	20.6	19.7	21.6	20.2	20.8
13	Title	13									20.4	20.1	22.0	20.7	19.9	21.2	20.0	20.8
14	Title	14									20.3	19.9	21.9	19.7	19.9	21.0	19.6	20.1
15	Title	15									20.2	20.2	21.3	19.9	20.2	21.0	19.6	20.1
16	Title	16									20.3	19.8	22.0	19.3	20.2	20.9	19.6	19.9
17	Title																	

Fig 4B-6

Table format: Survival		X	Group A	Group B
		DPI	Control	FiloCep vaccinat
		X	Y	Y
1	Title	6	1	
2	Title	6	1	
3	Title	6	1	
4	Title	6	1	
5	Title	6	1	
6	Title	7	1	
7	Title	7	1	
8	Title	7	1	
9	Title	28		0
10	Title	28		0
11	Title	28		0
12	Title	28		0
13	Title	28		0
14	Title	28		0
15	Title	28		0
16	Title			

Fig 4C-7

Table format: Grouped		Group A				Group B				Group C				Group D				Group E			
		Blood				Spleen				Liver				Kidney				Lung			
		A:Y1	A:Y2	A:Y3	A:Y4	B:Y1	B:Y2	B:Y3	B:Y4	C:Y1	C:Y2	C:Y3	C:Y4	D:Y1	D:Y2	D:Y3	D:Y4	E:Y1	E:Y2	E:Y3	E:Y4
1	V 3	1.0000	348.167400	1.000000	1.0000	1.0000	9400.000000	1.000000	1.00	1.00	1.00	1430.00	1.00	1.0000	1780.00	888.00	1.00	8490.00	844.00	2540.00	1840.00
2	C 3	1.8249e+009	3.437548e+009	2.299035e+008	3727629.0000	4.5000e+007	7.390000e+007	3.230000e+007	2.58e+007	3.67e+007	6.95e+007	2.49e+007	3.03e+007	3090000.0000	6160000.00	2810000.00	638000.00	3050000.00	1.99e+007	2000000.00	276000.00
3	V 5	1.0000	1.000000	38537.610000	1.0000	9450.0000	1770.000000	1.000000	1.00	126000.00	5430.00	6970.00	3720.00	2800.0000	1.00	1720.00	2800.00	4110.00	1050.00	1210.00	1.00
4	C 5	1.4071e+010				2.9500e+008	5.420000e+007	1.080000e+009		2.12e+009	4.18e+009	1.28e+009		4.0300e+007	4.47e+007	3.47e+008		1.44e+008	4.63e+007	3.01e+008	
5	Title																				

Fig 4C-8

		Group A Blood				Group B Spleen			
		A:Y1	A:Y2	A:Y3	A:Y4	B:Y1	B:Y2	B:Y3	B:Y4
1	V 3	1.0000	1.000000	1.000000	1.0000	1.0000	1.000000	1.000000	1.00
2	C 3	1050000.0000	2500000.000000	7340000.000000	199000.0000	2380000.0000	2.600000e+007	3830000.000000	1.37e+007
3	V 5	1.0000	1.000000	5880.000000	1.0000	1.0000	1.000000	1.000000	1.00
4	C 5	1.7200e+008	1.530000e+008	1.220000e+008	3.3800e+008	1710000.0000	2.460000e+007	1.140000e+007	7480000.00
5	Title								

		Group C Liver				Group D Kidney				Group E Lung			
		C:Y1	C:Y2	C:Y3	C:Y4	D:Y1	D:Y2	D:Y3	D:Y4	E:Y1	E:Y2	E:Y3	E:Y4
1	V 3	1.00	1.00	1.00	1.00	1.0000	1.00	1.00	1.00	1.00	1.00	1.00	1.00
2	C 3	4580000.00	6.95e+007	3610000.00	396000.00	45800.0000	202000.00	137000.00	163000.00	58700.00	539000.00	947000.00	104000.00
3	V 5	1.00	1.00	1.00	1.00	1.0000	1.00	1.00	1.00	1.00	1.00	1.00	1.00
4	C 5	5.70e+007	5.75e+007	7.24e+007	5.09e+007	657000.0000	2180000.00	1800000.00	2130000.00	4300000.00	6160000.00	2560000.00	2620000.00
5	Title												

Fig 5A-1

		Group A ChAdOx1.FILOcep1			Group B ChAdOx1.FILOcep2			Group C ChAdOx1.FILOcep1&2		
		A:Y1	A:Y2	A:Y3	B:Y1	B:Y2	B:Y3	C:Y1	C:Y2	C:Y3
1	P1	15	35	50	0	100	10	245	135	45
2	P2	0	0	110	0	0	0	45	0	15
3	P3	0	0	0	230	280	490	315	225	235
4	P4	1625	185	490	10	0	90	1505	685	875
5	P5	0	0	0	140	100	220	475	255	95
6	P6	0	0	130	30	0	30	325	135	0
7	P7	0	15	50	340	740	740	495	755	205
8	P8	135	305	80	0	90	70	315	155	125
9	P9	0	0	0	140	0	0	235	75	15
10	P10	0	0	0	0	120	10	65	105	65
11	P11	0	0	20	160	10	80	85	95	0
12	P12	35	0	40	10	0	10	35	75	0
13	Title									

Fig 5B-2

Table format: Grouped		Group A			Group B			Group C			Group D		
		Pool 3			Pool 4			Pool 5			Pool 7		
		A:Y1	A:Y2	A:Y3	B:Y1	B:Y2	B:Y3	C:Y1	C:Y2	C:Y3	D:Y1	D:Y2	D:Y3
1	M1	100	120	60	0	0	0	160	760	140		20	80
2	M2	120	720	180		180	160	80	140	160		40	660
3	M3	20	20	380	320	200	40	20	260	40		880	120
4	M4	40	60	320	80	160	540	780	40	820	40	620	680
5	Title												

Fig 5C-3

		X	Group A								Group B							
		DPI	Control								FILOcep1&2							
		X	A:Y1	A:Y2	A:Y3	A:Y4	A:Y5	A:Y6	A:Y7	A:Y8	B:Y1	B:Y2	B:Y3	B:Y4	B:Y5	B:Y6	B:Y7	B:Y8
1	Titl	1	21.5	19.9	19.8	21.9	23.3	20.7	24.2	19.6	21.4	21.1	23.4	22.2	21.5	22.7	20.0	18.6
2	Titl	2	21.7	20.3	20.3	22.5	23.4	20.9	24.3	20.2	22.0	21.3	23.1	22.6	22.0	22.6	20.4	19.2
3	Titl	3	21.1	20.2	20.1	21.7	23.1	20.1	23.1	19.7	22.0	21.7	22.4	22.2	21.7	22.7	20.0	19.3
4	Titl	4	20.9	18.8	18.4	20.8	21.9	18.6	22.2	19.0	22.5	21.9	22.6	22.5	21.8	22.4	20.6	19.0
5	Titl	5	19.8	19.0	18.3	20.0	21.8	18.2	21.6	18.0	22.5	22.0	22.8	22.6	22.1	22.6	20.8	21.0
6	Titl	6	18.1	17.4	16.6	18.3	19.9	16.7	19.9	16.8	22.3	22.4	22.7	23.2	22.9	22.1	21.0	19.7
7	Titl	7		17.2			19.5				22.7	22.7	23.5	23.6	23.7	23.7	22.3	20.5
8	Titl	8									22.5	22.1	22.8	23.3	22.2	22.3	21.4	20.1
9	Titl	9									22.4	21.8	22.0	22.9	22.4	23.1	21.7	19.7
10	Titl	10									22.3	21.6	22.2	23.0	22.5	23.7	21.5	20.0
11	Titl	11									21.6	21.8	22.4	23.8	22.7	24.0	21.3	20.6
12	Titl	12									22.4	22.2	23.3	23.5	22.2	21.5	22.0	20.6
13	Titl	13									21.6	22.6	23.2	23.1	22.7	22.9	21.6	21.4
14	Titl	14									22.0	22.8	23.6	23.1	23.1	24.0	22.1	20.9

Fig 5C-4

Table format: Survival		X	Group A	Group B	Gr
		DPI	Control	FILOcep1&2	
		X	Y	Y	
1	Title	7	1		
2	Title	7	1		
3	Title	7	1		
4	Title	7	1		
5	Title	7	1		
6	Title	8	1		
7	Title	8	1		
8	Title	6	1		
9	Title	28		0	
10	Title	28		0	
11	Title	28		0	
12	Title	28		0	
13	Title	28		0	
14	Title	28		0	
15	Title	28		0	
16	Title	28		0	
17	Title				

Fig 5C-5

		X	Group A								Group B								
		DPI	Control								FILOcep1&2								
		X	A:Y1	A:Y2	A:Y3	A:Y4	A:Y5	A:Y6	A:Y7	A:Y8	B:Y1	B:Y2	B:Y3	B:Y4	B:Y5	B:Y6	B:Y7	B:Y8	C
1	Titl	1	22.4	22.7	21.5	23.5	23.4	22.0	22.8	22.4	21.9	21.0	22.6	21.9	21.0	20.6	21.6	22.9	
2	Titl	2	22.4	22.3	21.6	23.0	22.8	20.1	22.3	22.5	22.0	21.6	22.4	22.0	21.5	20.8	21.8	22.9	
3	Titl	3	21.4	21.5	20.8	22.3	22.3	19.8	21.8	22.1	21.8	22.0	22.4	22.2	21.6	21.1	21.8	22.5	
4	Titl	4	21.1	20.8	20.3	20.5	21.7	18.9	20.9	20.5	22.5	21.7	22.0	22.5	21.4	20.7	21.6	22.8	
5	Titl	5	20.0	19.8	19.4	20.2	21.4	17.8	20.4	20.0	21.8	21.5	21.4	22.1	21.9	20.2	20.9	22.7	
6	Titl	6	18.5	18.3	18.3	17.9	20.1		19.8	20.0	22.1	21.9	22.1	22.1	21.6	20.9	20.6	23.3	
7	Titl	7	17.2	17.4		17.4	18.6		18.9	18.7	22.2	22.3	22.6	22.6	22.3	20.8	21.0	23.5	
8	Titl	8							18.7	18.0	22.5	22.1	22.2	22.7	22.4	20.6	21.9	23.0	
9	Titl	9							19.9		22.8	22.1	22.2	22.8	21.6	20.5	21.6	22.6	
10	Titl	10							20.4		22.5	22.3	22.3	22.6	21.8	20.6	21.6	22.9	
11	Titl	11							21.4		22.4	22.6	23.0	22.8	22.0	20.7	21.5	23.0	
12	Titl	12							22.1		22.1	22.2	22.9	23.1	22.6	21.3	21.3	23.4	
13	Titl	13							22.2		22.4	22.4	23.0	23.3	22.6	20.9	22.0	23.8	
14	Titl	14							21.6		22.6	22.7	23.1	24.1	22.7	21.7	22.8	24.0	
15	Titl																		

Fig 5C-6

		X	Group A	Group B	Gro
		DPI	Control	FILOcep1&2	T
		X	Y	Y	
1	Titl	8	1		
2	Titl	8	1		
3	Titl	8	1		
4	Titl	8	1		
5	Titl	7	1		
6	Titl	6	1		
7	Titl	9	1		
8	Titl	28	0		
9	Titl	28		0	
10	Titl	28		0	
11	Titl	28		0	
12	Titl	28		0	
13	Titl	28		0	
14	Titl	28		0	
15	Titl	28		0	
16	Titl	28		0	
17	Titl				
